# Dr. Mossman, on Digitalis

**Published:** 1800-04

**Authors:** Geo. Mossman

**Affiliations:** Bradford


### *To the Editors of the Medical and Phyfical Journal.*

Gentlemen,

In addition to the cafes which I have already detailed, I tranfmit to you the following. The preffure of arrangements neceffary to the publication of my Effay on *Scrophula* and *Confumption*, prevented me from furnifhing them for infertion in your laft Number.

Refpecting the curative powers of the *Digitalis*, there is a ftrange diverfity of opinion; and the evidence on both fides is refpectable: the bulk of practitioners, therefore, (by whom the qualities of the plant are certainly unknown) muft neceffarily be at a lofs, what belief they ought to efpoufe. As the fubject, however, has fo very generally arrefted medical attention, it will have an ample difcuffion; and it is much to be wifhed, that the public mind remain fufpended till experiment either eftablifh or fubvert the falutary effects of the *Digitalis*. When I firft entered upon the ufe of the plant in *Confumption*, I did not even entertain a hope that it could afford relief. I difbelieved its efficacy; and the iffue of the firft cafes, in which I prefcribed it, confirmed my fcepticifm. My patients perifhed! The failure I now afcribe to the circumftance of the plant being of a bad quality; fince its prominent effects were never never apparent. This confideration induces me to recommend the experimentalift to provide himfelf with the *genuine plant, properly collected, carefully preserved, and recently pulverized.* At prefent, we are certainly not employing the fame remedy, and of courfe the refult of our experience muft be widely different. Indeed, I have the ftrongeft conviction, that a want of due attention to the quality of the *Digitalis*, and to the quantity adminiftered, is the principal fource of medical difagreement relative to its powers. I am well perfuaded that this is the cafe; for I have recently feen fpecimens of the *Digitalis*, (fent to Apothecaries by their Druggifts, and warranted to be collected and prepared according to the moft approved method) the colour, flavour, and ftrength of which, were completely deftroyed by the application of too much heat. I have alfo known portions of the powder adminiftered, which had laid in a fhop-drawer for years. Are we to expect that the *Digitalis*, exhibited in this ftate, fhould exert its influence over the arterial fyftem, or produce any beneficial effect? The practitioner is difappointed; he abandons the further application of the plant; and he pronounces it ufelefs. I know fome ftriking inftances of this having happened; I therefore folicit the fcrupulous attention of medical men to the quality of the plant which they employ.

Again, I am perfuaded that the *Digitalis* is, in general, too fpeedily thrown into the fyftem, by dofes too large, and too frequently adminiftered. I am clearly of opinion, that in all cafes of *Pulmonary Confumption*, it fhould be fo managed, as not to difplay any of its powers previous to the termination of the firft, or commencement of the fecond week from its exhibition. I ought to obferve here, that there are cafes which require much time, and a large proportion of the plant, before they come under its influence; and I believe that thofe people who have indulged in the free ufe of fpirituous or ftrong liquors, are more difficultly affected than others. I have, however, met with none who did eventually refift its influence; and I think, that unlefs it obtain dominion over vafcular action, it will never produce any curative effect. It is therefore incumbent on every practitioner, who details failures, to ftate with precifion, whether the *Digitalis* did or did not retard the motion of the heart; for without this information we are not certain that their patients are charged with the plant; and the cafes are good for nothing. *I rejoice that the Digitalis is becoming a fafhionable remedy; and I know that the period will foon arrive, which will rank it with the firft of healing agents. In my mind, it is the moft important, and the moft extenfively ufeful inftrument in our art.*

In addition to what I have ſaid in my Eſſay, I have to obſerve, that I now keep my conſumptive patients on *animal food* and *cream* excluſively. I am,

GENTLEMEN,

Your's, obediently,

*Bradford, Feb.* 9, 1800. GEO. MOSSMAN.

### *CASES continued.*

CASE 10.—Sept. 5th. B. A. ætat. 27, caught cold about four weeks ago. He has fruitleſſly tried ſeveral remedies; he complains of a tightneſs and pain in his cheſt, accompanied by a violent dyſpnœa; his cough is hard, dry, and inceſſant; his tongue is furred; he has no appetite; his urine is high coloured; his pulſe is 120. I enjoined him to abſtain as much as poſſible from the uſe of liquids; and recommended his diet to conſiſt of *eggs* and *animal food*. I ordered a *bliſter* to be applied to the thorax; and preſcribed a grain of the *Digitalis* four times a day, with ſix drops of the *muriated barytes*.

Sept. 8th. The *bliſter* has operated well, and his breathing is eaſier; his cough is particularly diſtreſſing; his pulſe as frequent as before. There is yet no perceptible effect from the exhibition of the *Digitalis*.

Sept. 10th. His breathing is ſtill eaſier; his cough is more exaſperated than ever; his pulſe equally frequent. I diſcontinued the *muriated barytes*, and preſcribed the *pectoral julep* prepared as in caſe the 9th. I increaſed the doſe of the *Digitalis* to ſix grains a day.

Sept. 11th. He took ſix grains of the *Digitalis* yeſterday. He feels a ſenſation of giddineſs and ſtupor; his eyes are conſiderably affected; he ſeems to have a ſlight convulſive affection of the left ſide of his face; he vomited his breakfaſt; his pulſe is 110; his cough is eaſier; he experiences a load at his ſtomach, which is occaſionally accompanied by a faintneſs; I ordered him to day ſix grains of the *Digitalis* as before.

Sept. 12th. He feels relieved; he has vomited again this morning; he deſcribes a very ſingular ſenſation affecting his head and ſtomach; his pulſe is 90. I directed him to day to take only four grains of the *Digitalis*.

Sept. 15th. He vomits every morning; his urine continues high coloured, and he complains of a little pain when he voids it; he has pain in his head; and, when he awoke this morning, every object appeared to him as if covered with ſnow; this phænomenon continued for half an hour; rigors, ſucceſſive heats, and profuſe perſpirations follow each other; he expectorates pus, or thick mucus, occaſionally ſtreaked with blood; his pulfe is 100, with a confiderable intermiffion; his thirft is very great. I directed another *blifter* to be applied to the thorax; and I inftructed him to take only three grains of the *Digitalis* daily.

Sept. 18th. The *blifter* operated well; he continues to vomit occafionally; all objects ftill appear white at particular periods; his urine is become much paler; his pulfe intermits much, and is reduced to 68; his perfpirations are leffened; his cough is lefs diftreffing; and there appears a very complete alleviation of every morbid fymptom. He is obviously under the influence of the *Digitalis*. I inftructed him to continue its ufe as before.

Sept. 24th. He has been remarkably coftive, and has had recourfe to aperients; his tongue is clean, and his appetite is much improved; his cough and dyfpnœa have ceafed to give him any uneafinefs; and he can infpire fully without experiencing any painful fenfation in his cheft; he does not vomit; his vifion is natural; his expectoration is trifling; his pulfe is 80, regular and full; he feels an increafe of ftrength, and every confumptive fymptom is materially diminifhed. I directed him to take two grains of the *Digitalis* daily.

Oct. 13th. He called upon me in the moft perfect health; he faid his appetite was as good as it ever had been in his life, he had no complaint remaining, and had been following his ufual laborious employment for a week.

A few weeks afterwards this patient relapfed; without any inftructions from me, he again had recourfe to the *Digitalis*, and again it exhibited effects completely curative.

Case II.—Sept. 30th. T. H. ætat. 42, has been in a declining ftate of health for feveral months. About three weeks ago he contracted a fevere cold from fitting in wet cloaths; he was immediately feized with rigors, thirft, difficulty of breathing, and violent pain in his cheft. He cannot lie on his left fide, nor on his back, without exciting a moft diftreffing cough, and a fenfe of fuffocation. He expectorates a taftelefs white froth; his tongue is much furred; his urine is high coloured; his pulfe is 120. I enjoined a *milk* diet, and applied a *blifter* to the thorax; I alfo recommended the occafional employment of the *pectoral julep* already mentioned, and prefcribed four grains of the *Digitalis* daily.

Oct. 2d. The *blifter* operated well; his cough is much abated; and the dyfpnœa is very confiderably relieved. He can lie upon both fides without coughing, or experiencing any fenfe of fuffocation; his tongue is lefs furred; and his urine lefs high coloured. His pulfe is 90.

Oct. 7th. He has imprudently expofed himfelf to cold, which has produced a little tightnefs in his cheft, accompanied by a confiderable confiderable dyfpnœa and cough; his pulfe is 100. He continues to take four grains of the *Digitalis* daily.

Oct. 11th. He is much better; his pulfe is 65. I inftructed him to take two grains only of the *Digitalis* daily.

Oct. 15th. He is now in perfect health; and the further exhibition of the remedy appears unneceffary.

I was defired to vifit this patient again about the latter end of November, and found him labouring under an exacerbation of all his former fymptoms. He had enjoyed very tolerable health for feveral weeks, and had recovered his ufual ftrength. He had been from home upon bufinefs, and had been repeatedly wet, to which circumftance he afcribed the re-appearance of his complaints. I again had recourfe to the fame general method of cure, and prefcribed the *Digitalis*. I found, that he had, fince his former indifpofition, indulged much in the ufe of ftrong liquors; and that even at this period he could not be perfuaded to abandon their ufe. The *Digitalis* was taken, but never regularly; nor was he ever in any degree under its influence. A confirmed *pulmonary confumption* has continued to wafte him. He is now perifhing.

[ To be continued. ]

*N. B.* My next communication will contain a cafe of *mefenteric obftruction*, and fome cafes of *pleurify*, in which the falutary powers of the *Digitalis* were ftrikingly difplayed.

